# A Challenging Case of Managing Septic Arthritis of the Sternoclavicular Joint in a Patient With a History of Intravenous Opioid Use Disorder: A Case Report and Literature Review

**DOI:** 10.7759/cureus.42635

**Published:** 2023-07-29

**Authors:** Noman Khalid, Muhammad Adil Afzal, Safee Ullah Haider, Patrick Michael, Muhammad Abdullah

**Affiliations:** 1 Internal Medicine, St. Joseph's University Medical Center, Paterson, USA; 2 Internal Medicine, Shaikh Khalifa Bin Zayed Al-Nahyan Medical and Dental College, Shaikh Zayed Federal Postgraduate Medical Institute at Shaikh Zayed Medical Complex, Lahore, PAK; 3 Public Health and Community Medicine, Shaikh Khalifa Bin Zayed Al-Nahyan Medical and Dental College, Shaikh Zayed Federal Postgraduate Medical Institute at Shaikh Zayed Medical Complex, Lahore, PAK

**Keywords:** sternoclavicular joint (scj) septic arthritis, sternoclavicular joint (scj), iv drug use, opioid use disorder, septic arthritis

## Abstract

Septic arthritis of the sternoclavicular joint (SCJ) is a rare condition with limited literature available. We present a case of a 31-year-old female patient with a history of opioid drug use who presented with septic arthritis of the left SCJ. The patient exhibited chest wall pain; imaging revealed septic arthritis with an associated retrosternal abscess. Treatment with antibiotics alone resulted in the resolution of the abscess, highlighting the potential for medical management without surgical intervention. This case report and literature review emphasizes the importance of considering septic arthritis in patients with vague chest pain, particularly those with a history of intravenous drug use, and raise awareness about the complications associated with opioid use.

## Introduction

Septic arthritis of the sternoclavicular joint (SCJ) is a rare condition, with a prevalence estimated to be less than 1% of all septic arthritis cases [1,2]. Risk factors associated with this condition include immunosuppression, IV drug use, and diabetes mellitus [3]. *Staphylococcus aureus*, *Streptococcus* species, *E. coli*, and *Pseudomonas* are commonly reported causative agents [4-6].

Timely identification and immediate intervention are crucial since a delayed diagnosis could result in serious complications, such as mediastinitis, retrosternal abscess, chest wall phlegmon formation, osteomyelitis, and sepsis [7]. In contrast to septic arthritis of other joints, septic arthritis of the SCJ typically follows a more indolent course, making it challenging to detect on early plain radiographs. Therefore, MRI and CT imaging are considered more useful diagnostic tools [7]. The management of SCJ septic arthritis is controversial. While most authors advocate for surgical intervention in addition to medical management, the specific surgical approach and technique continue to be subjects of debate [8,9]. In this case report, we present a patient with a history of opioid drug use who presented with septic arthritis of the left SCJ. We also perform a literature review of similar cases in the literature.

## Case presentation

A 31-year-old female presented to the emergency department with left-sided upper chest wall pain that started two weeks ago. The pain was gradual in onset, rated 7 out of 10 in severity, dull in character, radiating to the left shoulder, exacerbated by movement of the left upper extremity, and not relieved with acetaminophen. The patient had a history of IV drug use, infective endocarditis, and methicillin-resistant *Staphylococcus aureus* (MRSA) bacteremia a few months ago, which had completely resolved. She had video-assisted lung surgery and lung decortication one year back. The patient reported injecting heroin into her neck, approximately 10 bags per day for a few years, with her last use being on the day of admission. Previously, the patient had injected drugs into both arms, resulting in an abscess formation that required drainage.

**Figure 1:**
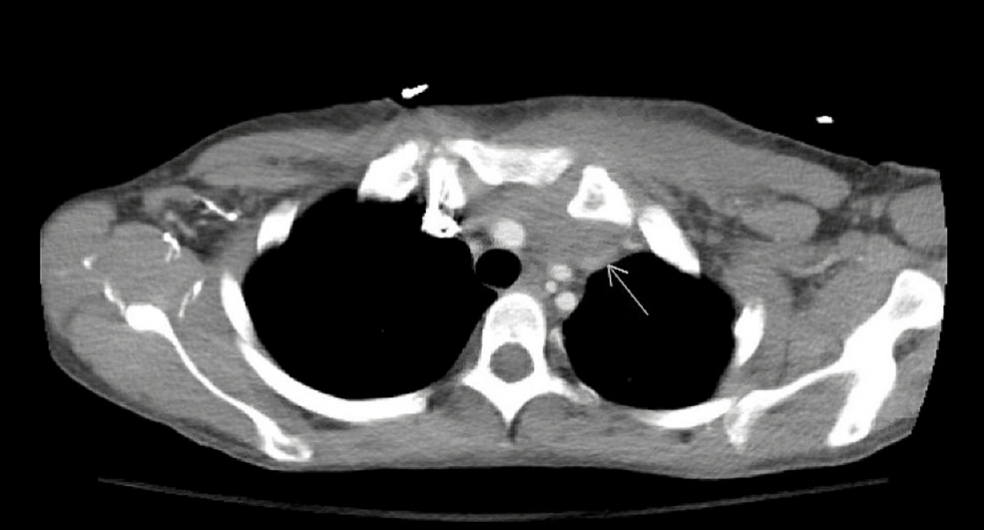
Contrast-enhanced axial CT scan of the upper chest showing soft tissue swelling, SCJ space widening with enlargement or thickening of the pectoralis muscle, and subcutaneous fat stranding

In the emergency department, the patient was hemodynamically stable with a blood pressure of 104/70 mmHg, a pulse of 68 bpm, and a temperature of 37 degrees Celsius. Physical examination revealed injection marks on the neck and bilateral upper and lower extremities and tenderness in the left upper chest wall. Laboratory results were significant for a lactic acid level of 3.2 mmol/L, a white blood cell count of 5700/mm3, an erythrocyte sedimentation rate of 73 mm/hr, a C-reactive protein level of 69.6 mg/L, deranged liver function tests including alkaline phosphatase of 158 U/L, aspartate transaminase of 609 U/L, and alanine transaminase of 802 U/L. The hemoglobin level was 11.6 mg/dL, and the troponin level was negative. Pneumonia and viral panel tests were negative, HIV 1 and 2 antibody screening was negative, hepatitis C virus (HCV) antibody was reactive, and quantitative, HCV PCR and Hep C RT-PCR showed 144000 IntUnit/ml, consistent with active infection. The hepatitis B surface antibody was positive, while the hepatitis B surface antigen and hepatitis B core antibody were negative, indicating immunity to hepatitis B. Rapid plasma reagin (RPR) was reactive with an RPR titer of 1:8 and a reactive fluorescent treponemal antibody absorption test. Urinalysis showed WBCs 6-10 high power field, trace leukocyte esterase, rare bacteria, and negative nitrites. Chest X-ray showed no active disease, and CT chest abdomen pelvis with contrast revealed septic arthritis of the left SCJ with erosion along the manubrium and a retrosternal collection (2.5 x 5.3 x 5 cm) communicating with the SCJ (Figure 1). The patient was admitted for left SCJ septic arthritis for further workup under the medical service. Treatment was initiated with IV vancomycin 1 g twice daily and cefepime 2 g every eight hours for septic arthritis. Infectious disease consultation was obtained for the management of septic arthritis and syphilis. Blood cultures were sent after antibiotic initiation and were negative. Cardiothoracic surgery (CTS) consultation was sought for the possibility of surgical collection drainage after initiating antibiotic therapy. Repeat CT thorax with contrast showed improvement in the swelling involving the left pectoral muscle and soft tissue thickening in the immediate retrosternal space on the left and one fragmentation of the adjacent cartilage and also irregularity of the manubrial junction suggestive of a focal erosion or cortical breakdown. Due to the reduction in the size of the swelling, CTS advised against surgical drainage and advised continuing with antibiotic therapy. As the abscess was not drained and no aspirate cultures were available, the specific organism causing the infection could not be identified.

During hospitalization, the patient began opioid withdrawal, and buprenorphine 8 mg three times a day was initiated as the patient's clinical opiate withdrawal scale (COWS) score was more than eight. Non-opioid adjunctive medications, including clonidine 0.1 mg oral, hydroxyzine 50 mg oral, and baclofen 5 mg oral, were administered as the patient's COWS score remained more than eight upon further assessment. Methadone 30 mg was initiated 24 hours after the last dose of buprenorphine for opioid abuse. Penicillin G 2.4 million units intramuscular once a week for three weeks was started to manage latent syphilis. Outpatient follow-up with gastroenterology and infectious disease specialists was recommended to control HCV infection. Upon stabilization, the patient was discharged with oral cefpodoxime 400 mg twice daily for 14 days, along with doxycycline 100 mg daily for 28 days to cover latent syphilis, as the patient was moving to a different state and would not be able to return for the remaining penicillin G doses.

## Discussion

Septic arthritis of the SCJ is a rare entity that can often go undiagnosed. Intravenous drug use is a risk factor for septic arthritis of the SCJ, which was also seen in our case [3]. Vague chest pain, particularly in patients with a history of intravenous drug use or immunosuppression, should be evaluated considering the possibility of septic arthritis of the SCJ, as the disease may lead to devastating complications. Our patient presented with a rare manifestation of the disease-an initial retrosternal abscess. The management of septic arthritis involves various modalities, including antibiotics alone or in combination with surgery.

While surgical abscess drainage is usually the preferred treatment, medical management was sufficient in our case. Although previous reports have documented the resolution of the condition with medical therapy alone, such cases are rare, especially in abscess formation, where surgical resection is typically preferred [10].

The literature review findings, as presented in Table 1, indicate a predominance of male patients (70%). The age range varied considerably, spanning from 25 to 81 years. CT scan was the most commonly used diagnostic modality. Notably, four out of 10 cases had a prior history of intravenous drug use. *Staphylococcus aureus* emerged as the most frequently isolated organism, identified in four cases. The management approaches were diverse, encompassing intravenous antibiotic administration, surgical resection, and debridement procedures. All cases in the literature review resolved with no complications.

**Table 1:** Cases of SCJ septic arthritis reported in the literature ESBL: extended-spectrum beta-lactamase, MRSA: methicillin-resistant *Staphylococcus aureus*, MSSA: methicillin-sensitive *Staphylococcus aureus*, IV: intravenous, CT scan: computed tomography scan, MRI: magnetic resonance imaging, IVDU: intravenous drug user, SCJ: sternoclavicular joint

Author, year	Age (years), sex	IVDU	Joint involved	Diagnostic investigation	Organism	Treatment
Present case	31, F	Yes	Left SCJ	CT scan with contrast	-	IV vancomycin, IV cefepime
Kim et al., 2022 [11]	38, F	No	Left SCJ	CT scan, culture of aspirate	Fusobacterium necrophorum	Piperacillin + tazobactam, amoxicillin + clavulanic acid, surgical exploration, and debridement
Alhariri et al., 2022 [12]	58, M	No	Left SCJ	MRI, blood culture	*E. coli* ESBL	IV ertapenem
Kraus, 2021 [13]	64, M	No	Right SCJ	MRI	MSSA	Cefazolin 2 g Q8H
Pinto and Schmitt, 2021 [14]	43, M	Yes	Right SCJ	Culture of joint aspirate, ultrasound, CT with contrast	MSSA	Intravenous antibiotics
Monteiro et al., 2021 [7]	58, M	No	Left SCJ	CT Scan, culture of aspirate	MSSA	Surgical exploration and debridement, IV linezolid, oral levofloxacin
Yeak et al., 2019 [15]	51, F	Unknown	Left SCJ	CT scan, blood culture	*Klebsiella* ESBL	IV meropenem
Shibayama et al., 2016 [4]	81, M	No		Culture of joint aspirate, ultrasound, CT with contrast	Streptococcus agalactiae	IV cefazolin
Barghi et al., 2010 [6]	25, M	Yes	Right SCJ	MRI, blood culture	MSSA	Intravenous antibiotics
Edelstein and McCabe, 1991 [16]	52, M	Yes	SCJ	Blood culture	Candida albicans	Surgical debridement, amphotericin B

Septic arthritis of the SCJ holds significant importance due to its association with IV drug use, particularly opioid use, which was declared a national emergency in the United States in 2017 [17]. Studies from 2015 to 2016 have reported 400,000 individuals reporting opioid use in the past month [18]. The projected annual number of opioid overdose deaths is expected to increase by 147% in 2025 compared to 2015 [19]. Given these circumstances, raising awareness about potential complications of opioid use, such as septic arthritis as reported in this case, is of paramount importance at the community level.

## Conclusions

Septic arthritis of the SCJ is a rare condition with potentially devastating complications, necessitating urgent management. The majority of cases are seen in IV drug users. Due to limited literature on the disease, there is no consensus on the general treatment for septic arthritis involving the SCJ. An approach consisting of antibiotics alone or a combination of antibiotics and surgical debridement may be employed. In our case, septic arthritis responded well to antibiotics alone. Therefore, based on the imaging and clinical presentation on a case-by-case basis, antibiotic therapy without surgical debridement may be appropriate for managing septic arthritis. Further reports of cases with similar presentations would provide additional evidence to establish a standardized treatment plan for this condition. The incidence of SCJ septic arthritis will likely continue to increase with the rising usage of IV drugs in the United States.

## References

[1] Gelberman RH, Menon J, Austerlitz MS, Weisman MH (1980). Pyogenic arthritis of the shoulder in adults. J Bone Joint Surg Am.

[2] Tapscott DC, Benham MD (2023). Sternoclavicular joint infection. https://www.ncbi.nlm.nih.gov/books/NBK551721/.

[3] Ross JJ, Shamsuddin H (2004). Sternoclavicular septic arthritis: review of 180 cases. Medicine (Baltimore).

[4] Shibayama A, Yoshizaki T, Tamaki M, Goto M, Takahashi T (2016). Pyogenic sternoclavicular arthritis caused by Streptococcus agalactiae in an elderly adult with diabetes mellitus. J Am Geriatr Soc.

[5] Vu TT, Yammine NV, Al-Hakami H, Hier MP, Black MJ (2010). Sternoclavicular joint osteomyelitis following head and neck surgery. Laryngoscope.

[6] Ghasemi Barghi R, Mirakbari SM (2010). Septic arthritis of sternoclavicular joint: a case report of a rare finding in injecting drug users. Arch Iran Med.

[7] Monteiro S, Gomes DS, Moura N, Sarmento M, Cartucho A (2021). Sternoclavicular septic arthritis: partial resection is still an option - a case report. J Orthop Case Rep.

[8] Ali B, Barlas V, Shetty AK, Demas C, Schwartz JD (2020). The preferred treatment of sternoclavicular joint infections: a systematic review. Cureus.

[9] Nusselt T, Klinger HM, Freche S, Schultz W, Baums MH (2011). Surgical management of sternoclavicular septic arthritis. Arch Orthop Trauma Surg.

[10] Kwon HY, Cha B, Im JH, Baek JH, Lee JS (2020). Medical management of septic arthritis of sternoclavicular joint: a case report. Medicine (Baltimore).

[11] Kim S, Kanwar R, Marshall MB (2022). Nonsurgical management of Fusobacterium necrophorum sternoclavicular septic arthritis: a case report. J Med Case Rep.

[12] Alhariri S, Kalas MA, Hassan M, Carter JT, Ghafouri SR, Dihowm F (2022). Medical management of septic arthritis of the sternoclavicular joint with extended-spectrum beta-lactamase-producing Escherichia coli: a case report. Cureus.

[13] Kraus J (2021). A complicated case of sternoclavicular septic arthritis. JAAPA.

[14] Pinto JF, Schmitt W (2021). Septic arthritis of the sternoclavicular joint in a patient with human immunodeficiency virus infection. Clin Case Rep.

[15] Yeak RD, Hussin P, Yap YY, Nizlan NM (2019). An atypical sternoclavicular septic arthritis that was treated conservatively. G Chir.

[16] Edelstein H, McCabe R (1991). Candida albicans septic arthritis and osteomyelitis of the sternoclavicular joint in a patient with human immunodeficiency virus infection. J Rheumatol.

[17] Strang J, Volkow ND, Degenhardt L (2020). Opioid use disorder. Nat Rev Dis Primers.

[18] Azadfard M, Huecker MR, Leaming JM (2023). Opioid addiction. https://www.ncbi.nlm.nih.gov/books/NBK448203/.

[19] Chen Q, Larochelle MR, Weaver DT (2019). Prevention of prescription opioid misuse and projected overdose deaths in the United States. JAMA Netw Open.

